# The TPx protein of *Taenia solium metacestode* regulates Treg and Th17 cell differentiation via the TGF-β/Smad signaling pathway

**DOI:** 10.3389/fimmu.2026.1612077

**Published:** 2026-02-06

**Authors:** Xiaoqing Sun, Qianqian Mu, Biying Zhou

**Affiliations:** 1 Department of Parasitology, Zunyi Medical University, Zunyi, China; 2 Binzhou Center for Disease Control and Prevention (Binzhou Key Laboratory of Emerging and Acute Infectious Diseases Research), Binzhou, China

**Keywords:** *Taenia solium metacestode*, TGF-β signaling pathway, Th17 cells, thioredoxin peroxidase, Treg cells, Treg/Th17 cell balance

## Abstract

**Introduction:**

The immunopathogenesis of cysticercosis remains elusive. This study investigates the effects of *Taenia solium* metacestode-derived thioredoxin peroxidase (TPx) protein on regulatory T (Treg) cells and T helper 17 (Th17) cell differentiation, as well as its correlation with signaling pathways in human Jurkat T lymphocytes exposed to TPx for varying durations, to provide a scientific basis for further studying the immune pathogenesis and clinical treatment of cysticercosis.

**Methods:**

TPx protein from the excretory-secretory antigens of *T. solium metacestode* was used to stimulate Jurkat cells at various timepoints. Flow cytometry was employed to detect the expression of CD4^+^CD25^+^CD127^-^ Treg cells and CD4^+^IL-17A^+^ Th17 cells. Transcriptomic analysis was performed to identify the signaling pathways related to the differentiation of Jurkat cells under the influence of the TPx protein at different time points. The expression levels of TGF-β1, TGF-βR2, Smad4, Foxp3, RORC(γt), and phosphorylated Smad3 proteins were measured in TPx-treated cells and control cells at different time points.

**Results:**

A Treg/Th17 cell imbalance was detected in Jurkat cells following exposure to TPx, characterized by a Treg-mediated predominant immunosuppressive response. Gene Set Enrichment Analysis identified significant enrichment of the TGF-β signaling pathway and Th17 cell differentiation pathway in TPx-treated cells. TPx significantly upregulated TGF-β1, TGF-βR2, and p-Smad3 expression in cells (*P* < 0.05). Concurrently, the expression of Foxp3, a key transcriptional regulator of Treg cell differentiation, was markedly increased (*P* < 0.05). In contrast, the expression of RORC(γt), a transcription factor critical for Th17 cell differentiation, showed significant reduction after 72 h of TPx induction (*P* < 0.05).

**Discussion:**

The TGF-β/Smad signaling pathway is a crucial molecular mechanism involved in the imbalance of Treg/Th17 cells induced by the TPx protein of *T. solium metacestode*.

## Graphical Abstract

**Figure:**
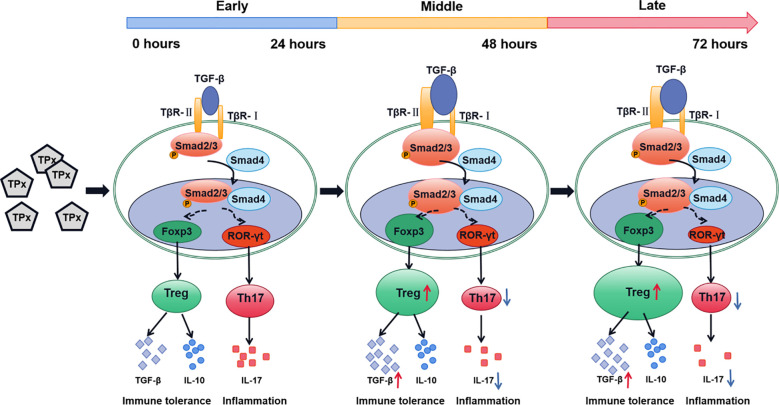
The TPx protein of Cysticercus cellulosae regulates the expression levels of Foxp3 and RORC (γt) by activating the TGF-β/Smad signaling pathway. Early (24 h) differentiation of Treg cells is slightly inhibited, while differentiation of Treg cells is promoted and differentiation of Th17 cells is inhibited in the mid to late stages (48 h, 72 h). This leads to an imbalance of host Treg/Th17 cells, resulting in an immune response predominantly characterized by Treg cells. This study primarily investigates the effects of the TPx protein on the differentiation of Treg and Th17 cells and its correlation with the TGF-β/Smad signaling pathway, providing important scientific evidence for further exploration of the immunopathogenesis of Cysticercosis.

## 1 Introduction

The pork tapeworm *Taenia solium* (*T. solium*) is a neglected zoonotic parasite that ranks first globally among parasitic causes of foodborne infections (1). Infections between humans and pigs perpetuate a vicious cycle (2). The severity of cysticercosis depends on the number of parasites and their location. According to the World Health Organization (WHO), neurocysticercosis (NCC) is considered a neglected tropical disease that affects approximately 50 million people globally, causing 50,000 deaths annually (3). Current treatments primarily involve medications and surgical interventions, both of which have limitations. The complex immune pathogenesis induced by *T. solium metacestode* is a significant barrier to the development of effective vaccines (4, 5). Therefore, elucidation of the host immune regulation induced by *T. solium metacestode* is critically warranted.

CD4^+^ T cells play a central role in anti-parasitic immunity, where the dynamic balance between Treg and Th17 cells is crucial for determining infection outcomes (6, 7). Our previous work demonstrated that excretory-secretory antigens (ESAs) from *T. solium* metacestodes can modulate host immune responses by activating the TGF-β/Smad signaling pathway (8). The TGF-β/Smad signaling pathway is a key regulator of T cell fate. Upon binding to its receptors (TβR-I/II), TGF-β activates Smad2/3 proteins, which then translocate to the nucleus (9–12). There, they modulate the expression of lineage-specific transcription factors, such as Foxp3 for Treg cells and ROR-γt for Th17 cells, thereby critically directing their differentiation (13; 14). Notably, this pathway is often exploited by parasites to manipulate host T cell responses and promote immune evasion. Through proteomic analysis, we further identified the thioredoxin peroxidase (TPx) protein from these antigens and confirmed that TPx could induce Treg/Th17 imbalance in piglets, thereby facilitating immune evasion (15, 16). The TPx protein of *T. solium* metacestode exhibits high sensitivity and specificity, making it a specific antigen for the diagnosis of cysticercosis and a potential candidate for vaccine development for immunoprophylaxis against this disease (17). However, the precise molecular mechanisms by which TPx regulates the differentiation of these cell subsets remain unclear.

Building on these findings, this study aimed to investigate the effect of *T. solium* metacestode TPx protein on the differentiation of human Treg and Th17 cells and to elucidate the underlying mechanisms. Using a Jurkat cell model, we analyzed the regulatory role of TPx on the differentiation of both cell types at different time points. Furthermore, we employed transcriptomics, flow cytometry, and Western blotting to verify the key involvement of the TGF-β/Smad signaling pathway in this process. Our work provides new experimental evidence for clarifying the mechanism of TPx-mediated immune evasion.

## 2 Materials and methods

### 2.1 Preparation of *T. solium metacestode* TPx protein

Bioinformatics analysis was employed to predict the hydrophilicity and T cell antigenic epitopes of TPx protein. The TPx antigen-coding gene was synthesized using whole-gene synthesis technology. The gene was then cloned into the pcDNA3.4 vector to construct the recombinant plasmid pcDNA3.4-TPx. The recombinant plasmid was enzymatically digested and sequenced for verification. The plasmid was transfected into Expi 293 cells and the expressed TPx protein was purified using a Ni column (Endotoxin<10 EU/mg). The purified protein was analyzed by SDS-PAGE and further confirmed by western blotting. The recombinant TPx protein was stored at −80°C.

### 2.2 Cultivation and activation of Jurkat cells

The Jurkat T cell model can simulate human T lymphocyte functions, and is widely used in *in vitro* studies of T cell signal transduction, cytokine production, and receptor expression. It also provides significant guidance and reference for the treatment and mechanistic research of various infectious diseases (18). Human leukemia T lymphocyte Jurkat cells, Clone E6-1 (hereinafter referred to as “Jurkat cells”), were purchased from the Shanghai Cell Bank of the Chinese Academy of Sciences. Cells from suspension culture were cultured in RPMI 1640 medium (Solarbio Sciences, China) containing 10% fetal bovine serum (Gibco, Australia) and antibiotics (100 μg/ml penicillin and 100 μg/ml streptomycin). Cells were treated with 0.5 ng/ml Phorbol-12-myristate-13-acetate (PMA) and 0.5 μM ionomycin for 12 hours to induce cell activation. Based on this, 500 ng/ml of TPx protein was used according to the schedule set for each experimental cell group.

### 2.3 Flow cytometry analysis

Activated Jurkat cells were washed with pre-cooled PBS (Meilunbio, China) and then treated with TPx protein (500 ng/mL) for 24 h, 48 h, and 72 h, while control cells were cultured with an equal volume of RPMI 1640 medium instead of TPx protein. All cells were cultured under standard conditions in 6-well plates at a density of 1×10^6^ cells/mL. Cells from each culture group were collected separately, counted, and set aside for later use. We collected a total of 1 × 10^6^ cells and washed them with pre-cooled PBS (Meilunbio, China). One group was incubated with appropriate amounts of allophycocyanin (APC)-labeled anti-human CD4 antibody (BD Pharmingen, USA), phycoerythrin (PE)-labeled anti-human CD25 antibody (BD Pharmingen, USA), and fluorescein isothiocyanate (FITC)-labeled anti-human CD127 antibody (Thermo Fisher, USA). The cells were mixed well and incubated on ice in the dark for 30 minutes. Another group was incubated with an appropriate amount of APC-labeled anti-human CD4 antibody (BD Pharmingen, USA), mixed well, and incubated on ice in the dark for 30 minutes. After fixation and membrane permeabilization (After fixation and membrane permeabilization, USA), an appropriate amount of peridinin-chlorophyll-protein complex-cyanine5.5 (PerCP-Cy5.5)-labeled anti-human IL-17A antibody (Thermo Fisher, USA) was added. The cells were mixed well and incubated on ice in the dark for 20 minutes. We then washed the cells and resuspended them in PBS for flow cytometry analysis (BD FACSAria III, USA).

### 2.4 Transcriptome sequencing and bioinformatic analysis

Activated Jurkat cells were washed with pre-cooled PBS (Meilunbio, China) and then treated with TPx protein (500 ng/mL) for 0 h, 48 h, and 72 h, while control cells were cultured with an equal volume of RPMI 1640 medium instead of TPx protein, each experimental group consisted of three samples. Jurkat cells were cultured in a 6-well plate at a density of 1 × 10^6^ cells/well, at 37°C with 5% CO_2_. After the completion of Jurkat cell culture, the cells were centrifuged at 1500 g for 10 min at 4°C to remove the culture medium. Then, the cells were resuspended in PBS bufer (Solarbio Sciences, China) and centrifuged again at 1500 g for 10 min at 4°C, followed by a quick wash. The cell pellets were resuspended in TRIzol (Takara, Japan) at a ratio of 1 ml per 1 × 10^6^ cells and vigorously pipetted until a clear and non-viscous liquid was formed. The lysate was then transferred to a new centrifuge tube. The extracted RNA samples were sent to Shanghai OE Biotech Co., Ltd. for quality inspection. After meeting the requirements, library construction was performed, and the library quality was evaluated. Finally, RNA-seq sequencing and sequence alignment were conducted with the assistance of Shanghai OE Biotech Co., Ltd.

The steps for constructing a transcriptome sequencing library include total RNA extraction, RNA quality assessment and fragmentation, reverse transcription to generate cDNA, end repair, A-tailing, adapter ligation, and PCR amplification, followed by RNA-seq sequencing and sequence alignment. Based on the transcriptome sequencing results, DESeq2 software was used with *p*-value < 0.05 and fold change (FC) > 2.0 or < −2.0 as criteria for significant differences, to identify differentially expressed protein-coding genes for Gene Ontology (GO) functional analysis, Kyoto Encyclopedia of Genes and Genomes (KEGG) pathway enrichment, and Gene Set Enrichment Analysis (GSEA) enrichment analysis.

### 2.5 Western blotting

Jurkat cells from each culture group were collected and resuspended in pre-cooled PBS for washing. Cells were then lysed in freshly prepared Radio-Immunoprecipitation Assay lysis buffer (Solarbio Sciences, China) on ice for 30 minutes, followed by centrifugation at 12,000 rpm for 20 minutes at 4 °C. The supernatant was aspirated to obtain total cellular protein. Sample buffer was added, and the proteins were denatured by boiling for subsequent experiments (19).

After protein electrophoresis and membrane transfer, the membranes were blocked by soaking in 5% non-fat milk for 2 hours, then washed three times with Tris-Buffered Saline with Tween (TBST) buffer for 10 minutes each. The membranes were incubated with diluted primary antibodies against TGF-β1 (Thermo Fisher, USA), TGF-βR2 (Proteintech, China), Smad4 (Proteintech, China), Foxp3 (Thermo Fisher, USA), RORC(γt) (Proteintech, China), Smad3 (Thermo Fisher, USA), and p-Smad3 (Abcam, UK) on ice for over 12 hours. After incubation, the membranes were washed three times with TBST buffer for 10 minutes each. Goat anti-rabbit secondary antibody (Proteintech, China) was prepared in TBST buffer according to the antibody dilution ratio and incubated at room temperature for 1.5 hours. Finally, the membranes were washed three times with TBST buffer for 10 minutes each, and the proteins were detected using ECL reagent (Meilunbio, China).

### 2.6 Statistical analysis

We used GraphPad Prism 8 for data visualization and SPSS 29.0 for statistical analysis. Data are expressed as mean ± standard deviation (x̄ ± SD, n = 3) using the independent sample t-test. *P* < 0.05 was considered to indicate statistical significance.

## 3 Results

### 3.1 The effect of *T. solium metacestode* TPx protein on the differentiation of Treg and Th17 cells

**Figure 1:**
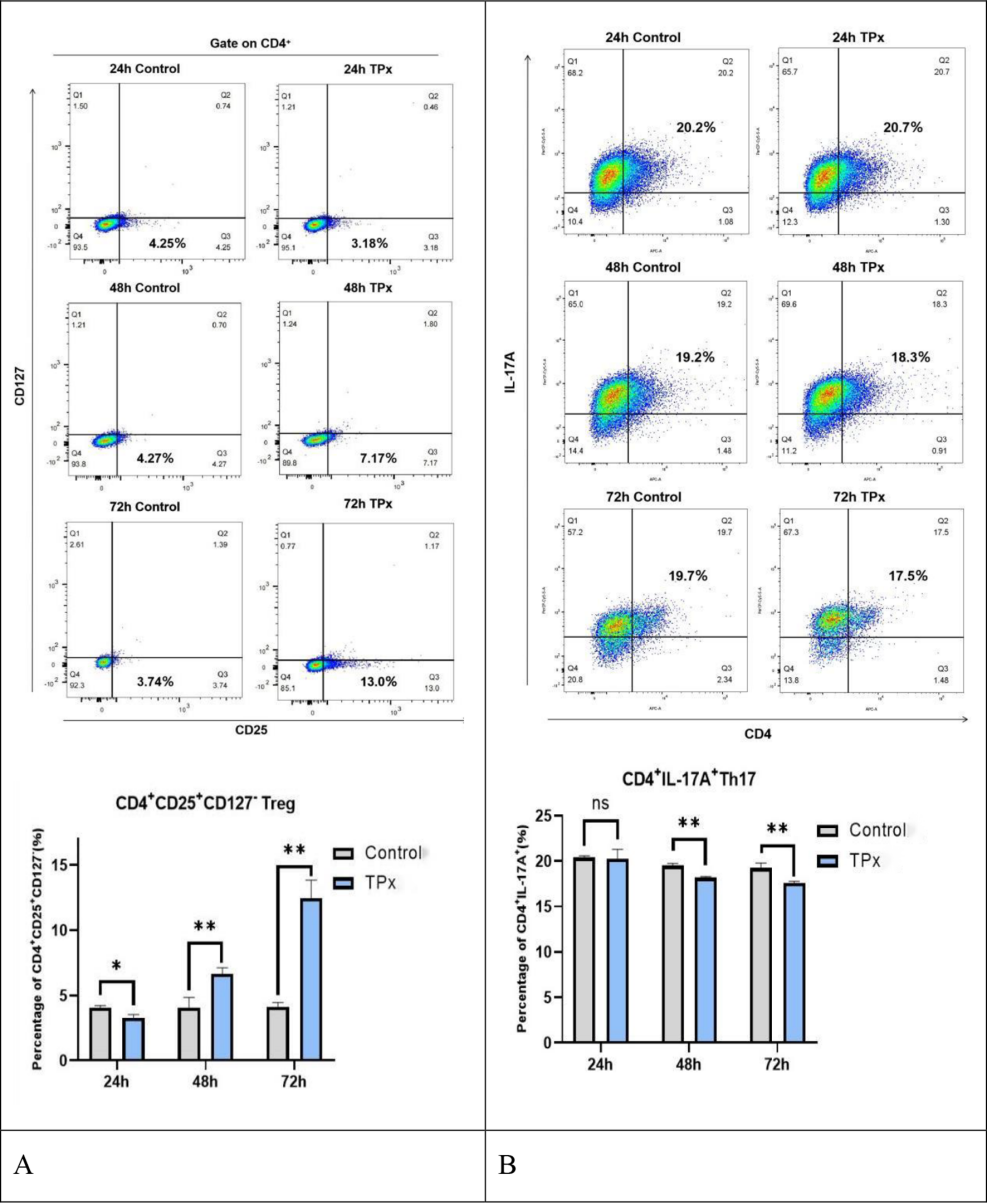
Effect of TPx protein on the differentiation of Treg and Th17 cells (n = 3, x̄ ± S, %). **(A)** Effect of TPx protein on Treg cell differentiation; **(B)** Effect of TPx protein on Th17 cell differentiation; *P < 0.05; **P < 0.01; ns, no significant difference. The Treg/Th17 ratio was analyzed in Jurkat cells after treatment with TPx. Compared with the control cells, there was no significant difference after 24 h of TPx protein treatment. However, after 48 h and 72 h, TPx protein significantly induced an imbalance between Treg and Th17 cells. With increasing duration of stimulation, the ratio of Treg/Th17 cells exhibited an upward trend, resulting in a predominantly Treg cell-driven inhibitory immune response.

TPx protein was applied to Jurkat cells for 24 h, 48 h, and 72 h. Control cells was included that did not receive TPx protein. The expression of CD4^+^CD25^+^CD127^−^ Treg cells in each sample was assessed using flow cytometry to examine the impact of TPx protein on Treg cell differentiation over time. As shown in **Figure 1**, compared with the control cells, TPx protein inhibited Treg cell differentiation at 24 h, with a significant difference (*P* < 0.05), while its effect on Th17 cells was not significant. However, after 48 h and 72 h of TPx protein treatment, there was a significant induction of Treg cell differentiation and inhibition of Th17 cell differentiation (*P* < 0.05).

**Figure 2:**
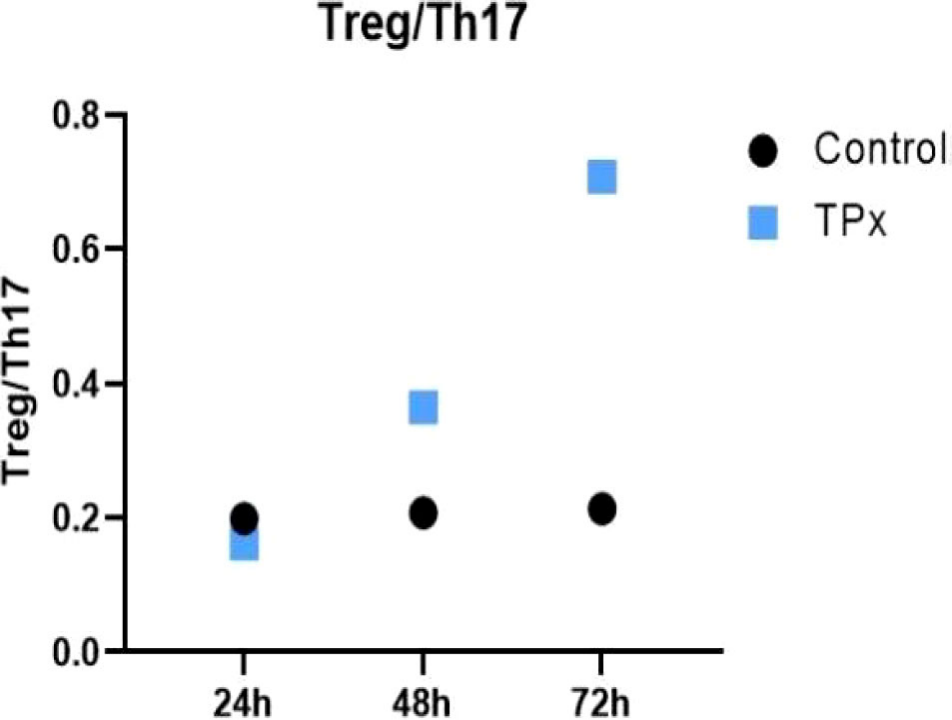
Effect of TPx protein on the balance of Treg/Th17 cells. The black dots represent the ratio of the CD4^+^CD25^+^CD127^-^ Treg cell subset to the CD4^+^IL-17A^+^ Th17 cell subset in the control cells at different time points; the blue squares represent the ratio of the CD4^+^CD25^+^CD127^-^Treg cell subset to the CD4^+^IL-17A^+^ Th17 cell subset in the TPx-treated cells at different time points.

Treg/Th17 ratio was analyzed in Jurkat cells after treatment with TPx. As shown in **Figure 2**, compared to the control cells, there was no significant difference after 24 h of TPx protein treatment. However, after 48 h and 72 h, TPx protein significantly induced an imbalance between Treg and Th17 cells. With increasing stimulation time, the ratio of Treg/Th17 cells exhibited an upward trend, resulting in a predominantly Treg cell-driven inhibitory immune response.

### 3.2 Differential analysis of genes expression in human Jurkat T cells upon C. cellulose TPx protein treatment

#### 3.2.1 Differential expression gene analysis

**Figure 3:**
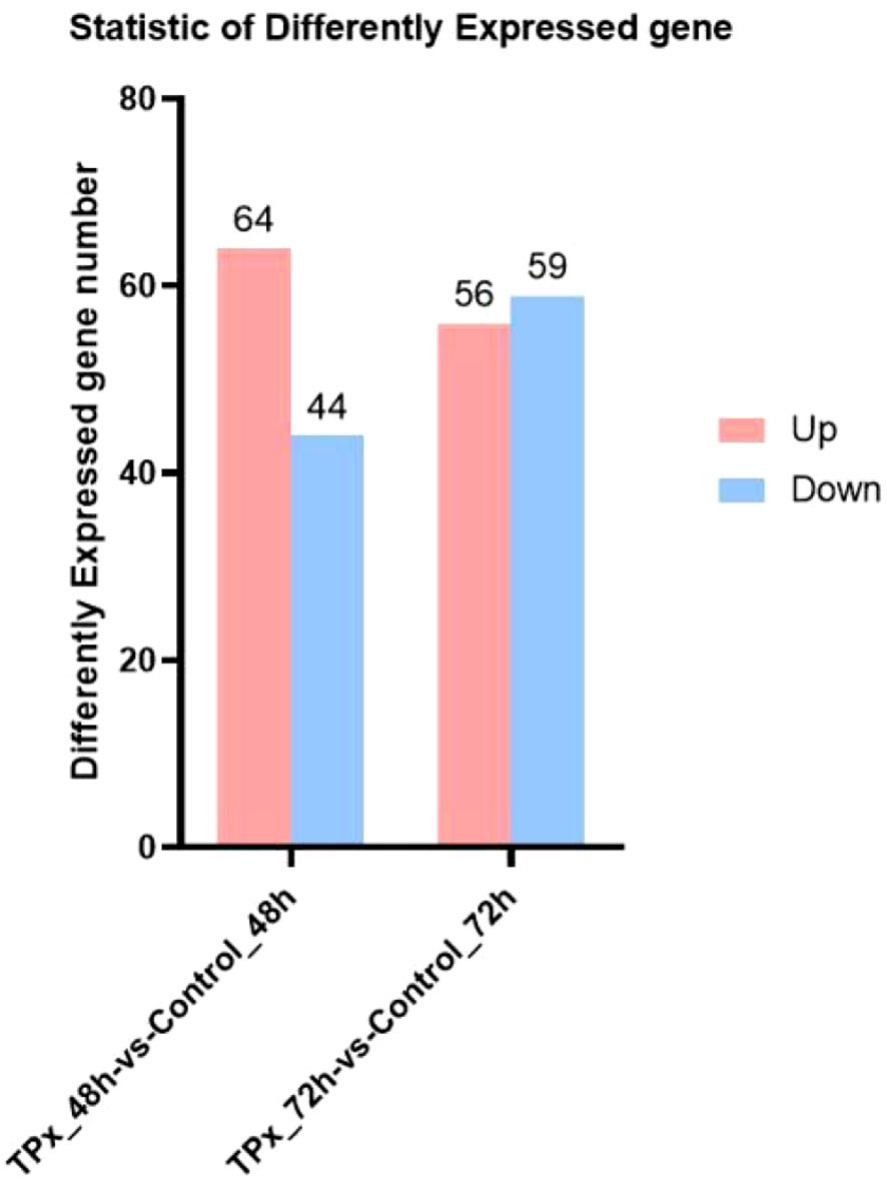
Bar graph of differentially expressed genes.

**Figure 4:**
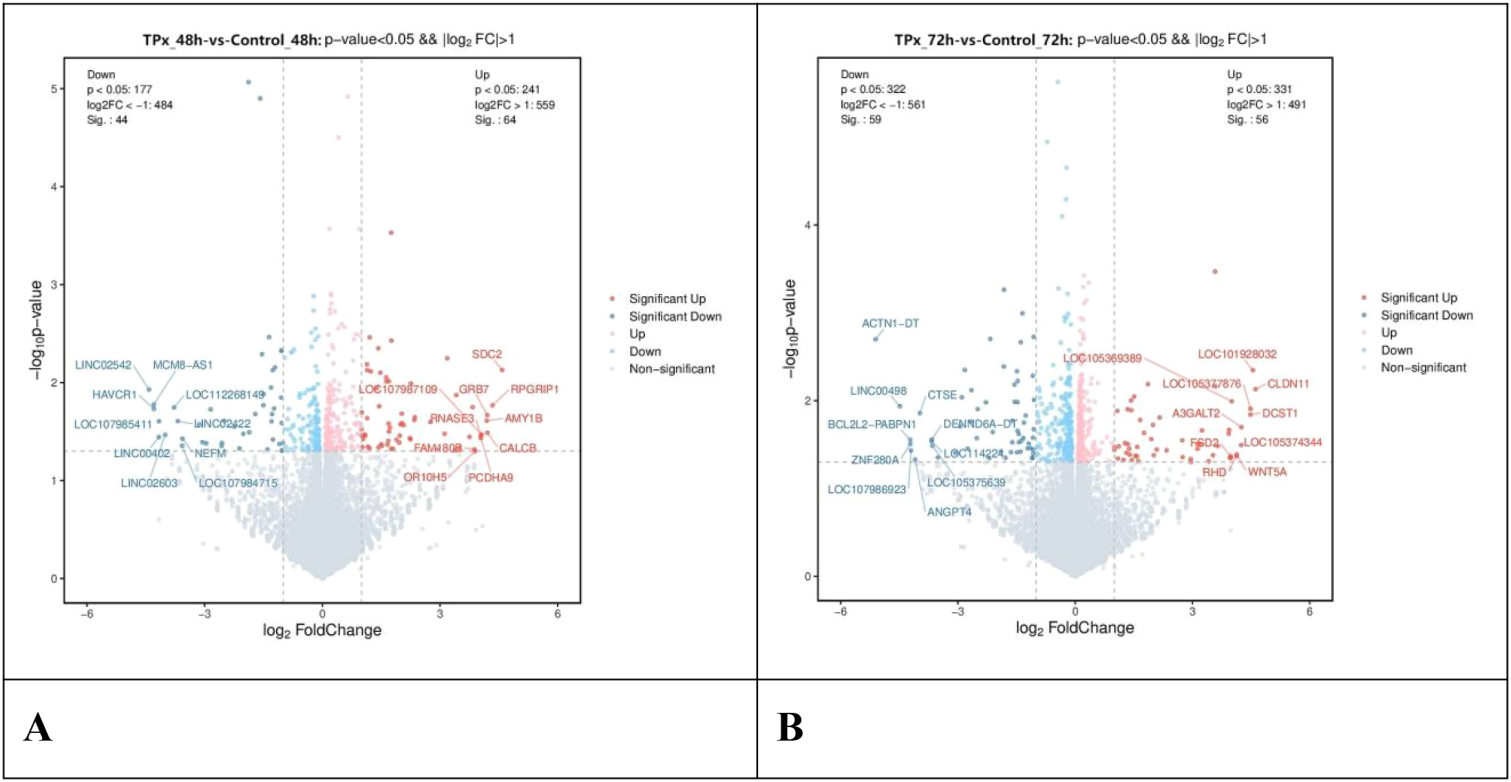
Volcano plot comparing differentially expressed genes between the TPx-treated cells and the control cells. **(A)** upon 48 h treatment; **(B)** upon 72 h treatment; Dots in the plot represent individual genes, arranged on the x-axis based on the logarithm of the expression ratio between groups and on the y-axis based on the statistical significance of the difference in expression between groups (FDR/adjusted p-value). The absolute value of the log_2_ expression ratio of 1 (min. 2-fold higher gene expression in one or another group of samples) was taken as the significance threshold on the x-axis, and a -log_10_ p-value of less than 0.05 (*p* < 0.05) was set as the significance threshold on the y-axis. Genes without significant differences are shown in gray, while significantly differentially expressed genes are color-coded in red and blue. Specifically, red denotes upregulated genes and blue denotes downregulated genes. The greater the distance from the dashed line, the more pronounced the difference in gene expression between the two groups.

As shown in the **Figures 3** and **4**, statistical analysis revealed that compared to the 48 h control cells, a total of 108 genes with significant differential expression were identified in the 48 h TPx-treated cells. Among these, 64 genes were upregulated, including primarily SDC2, RPGRIP1, and AMY1B; 44 genes were downregulated, including primarily HAVCR1, NEFM, and LINC02603. Compared to the 72 h control cells, a total of 115 genes with significant differential expression were identified in the 72 h TPx-treated cells. Among these, 56 genes were upregulated, including primarily FSD2, CLDN11, and WNT5A; 59 genes were downregulated, including primarily ZNF280A, ACTN1-DT, and CTSE.

#### 3.2.2 GO functional annotation

**Figure 5:**
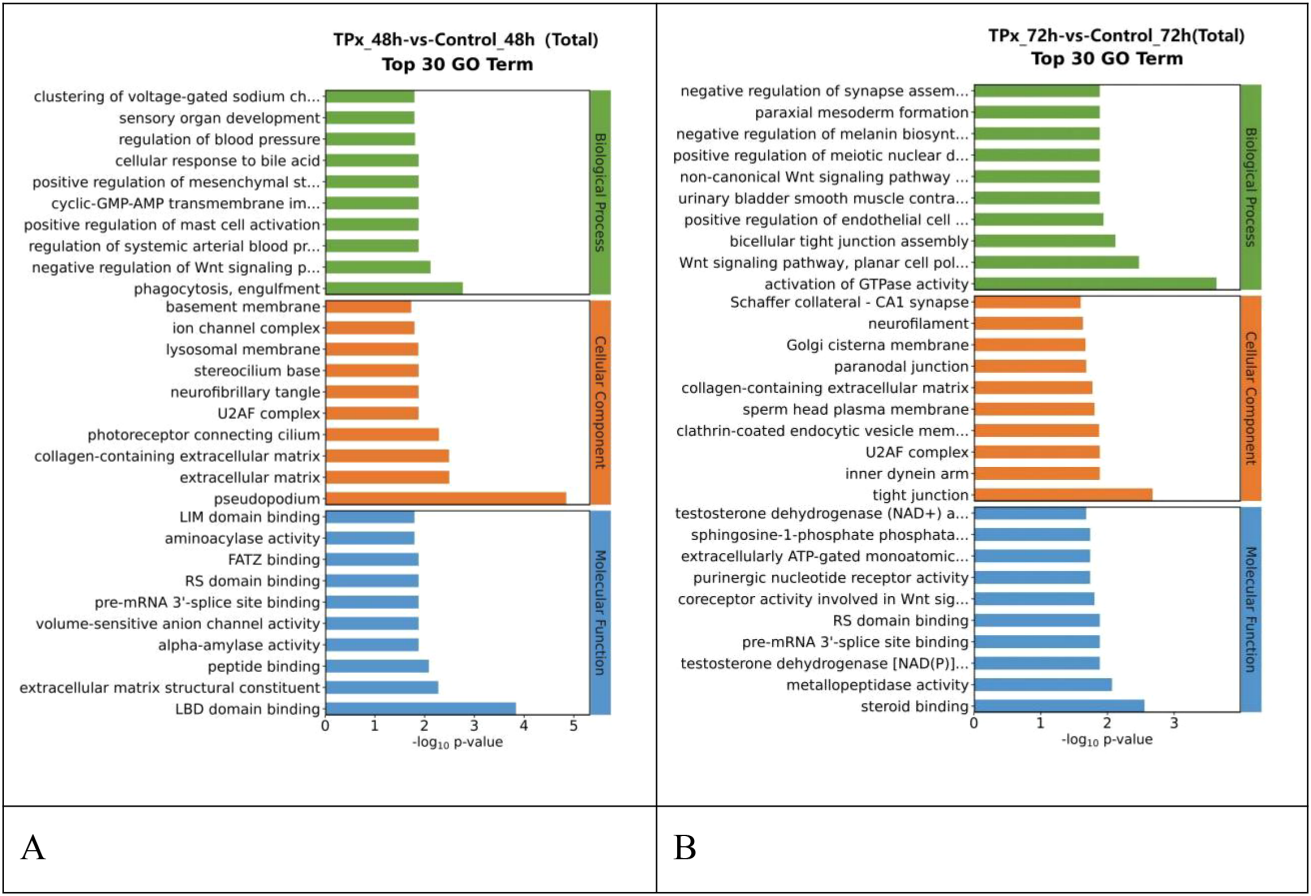
Top 30 enriched GO terms between the TPx-treated cells and the control cells. **(A)** Top 30 enriched GO terms between the 48 h TPx-treated cells and the 48 h control cells; **(B)** Top 30 enriched GO terms between the 72 h TPx-treated cells and the 48 h control cells; The horizontal axis represents -log_10_ p-values, and the vertical axis represents enriched GO terms.

As shown in the **Figure 5**, the GO terms enriched in the differential genes between the 48 h TPx-treated cells and the 48 h control cells mainly include: phagocytosis, extracellular matrix structural constituent, and positive regulation of mast cell activation. The GO terms enriched in the differential genes between the 72 h TPx-treated cells and the 72 h control cells mainly include: activation of GTPase activity, Wnt signaling pathway, cell polarity pathway, and formation of two-cell tight junctions. These findings indicate that TPx protein can induce changes in T cell immune responses, cell differentiation, and signaling molecules in Jurkat cells.

#### 3.2.3 Enrichment analysis of KEGG signaling pathways

**Figure 6:**
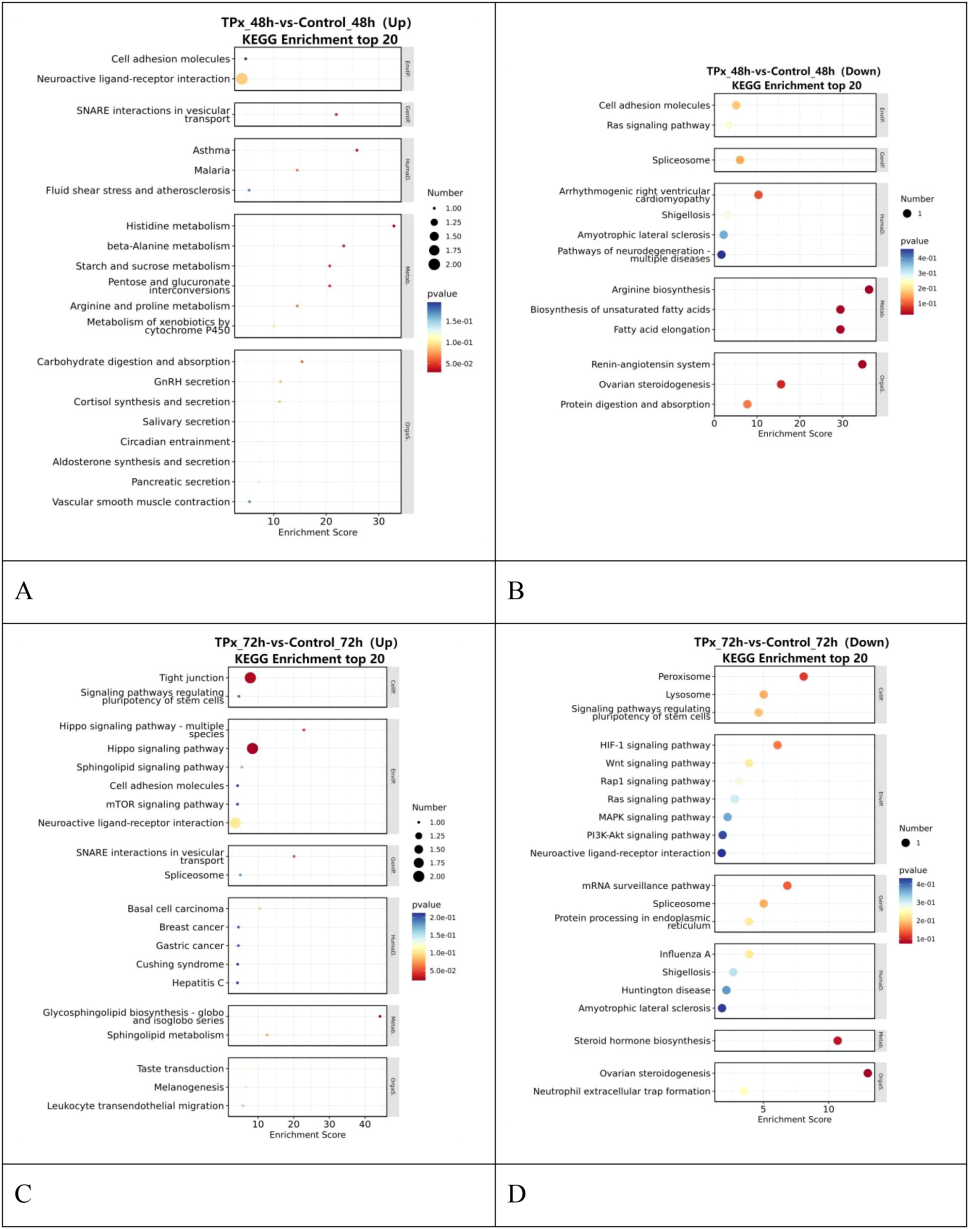
Bubble plot comparing KEGG pathways between the TPx-treated cells and the control cells. **(A)** upon 48 h of TPx protein treatment (Up); **(B)** upon 48 h of TPx protein treatment (Down); **(C)** upon 72 h of TPx protein treatment (Up); **(D)** upon 72 h of TPx protein treatment (Down). Top 20 pathways are shown, with enrichment scores and p-values indicated by dot size and color.

As shown in **Figures 6A, B**, compared with the 48 h control cells, the 48 h TPx-treated cells experimental group were mainly enriched in upregulated signaling pathways including cell adhesion molecules, neuroactive ligand-receptor interaction, and histidine metabolism. The downregulated pathways primarily include Ras signaling pathway, spliceosome, and protein digestion and absorption. As shown in **Figures 6C, D**, compared with the 72 h control cells, the 72 h TPx-treated cells were mainly enriched in upregulated signaling pathways such as the Hippo signaling pathway, cell adhesion molecules, and neuroactive ligand-receptor interaction. The downregulated pathways primarily include the HIF-1 signaling pathway, Ras signaling pathway, mitogen-activated protein kinase (MAPK) signaling pathway, and PI3K-Akt signaling pathway. The results indicate that these differentially expressed genes are primarily involved in processes such as the cell cycle, cellular biological responses, and T cell proliferation and differentiation.

#### 3.2.4 GSEA enrichment analysis

**Figure 7:**
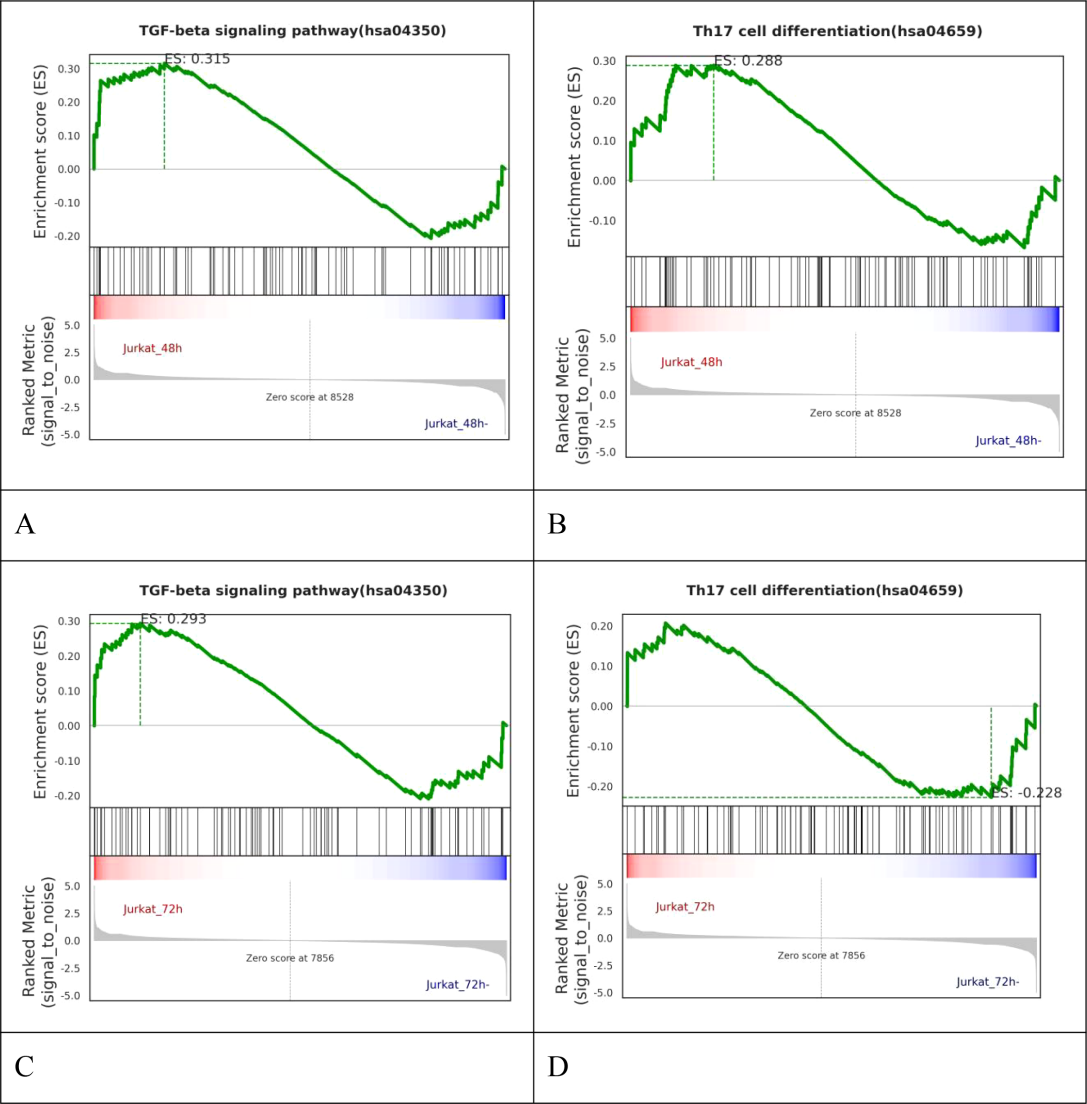
Enrichment plot of GSEA pathways. **(A)** Enrichment plot of GSEA pathways for the TGF-β signaling pathway comparing the 48 h TPx-treated cells with the 48 h control cells; **(B)** Enrichment plot of GSEA pathways for Th17 cell differentiation comparing the 48 h TPx-treated cells with the 48 h control cells; **(C)** Enrichment plot of GSEA pathways for TGF-β signaling pathway comparing the 72 h TPx-treated cells with the 72 h control cells; **(D)** Enrichment plot of GSEA pathways for Th17 cell differentiation comparing the 72h TPx-treated cells with the 72 h control cells; the figure is divided into four parts from top to bottom: ① Distribution of Enrichment Scores (ES), where the green line represents the distribution of ES for all genes. The peak of this curve corresponds to the absolute maximum ES value for the gene set. When ES > 0, core genes are on the left side of the peak; when ES < 0, they are on the right side. ② Gene Distribution Plot, where vertical lines represent the positions of genes in the entire ranking. ③ Colorbar, which is the color mapping of the ranking matrix. Positive values correspond to red, where larger values are deeper red, and negative values correspond to blue. Values closer to 0 are closer to white. ④ Distribution Plot of the ranking matrix, showing fold change, signal-to-noise ratio, etc. **(A)** GSEA pathway enrichment plot for the TGF-β signaling pathway; **(B)** GSEA pathway enrichment plot for Th17 cell differentiation.

To explore the signaling pathways through which the *T. solium metacestode* TPx protein affects the differentiation of Treg/Th17 cells, based on the KEGG database, further GSEA was performed for signaling pathway enrichment analysis on differential genes. As shown in **Figures 7A, B**, compared with the 48 h control cells, the TGF-β signaling pathway and Th17 cell differentiation pathway were upregulated in the 48 h TPx-treated cells. As shown in **Figures 7C, D**, compared with the 72 h control cells, the TGF-β signaling pathway was upregulated in the 72 h TPx-treated cells, while the Th17 cell differentiation pathway was downregulated.

**Figure 8:**
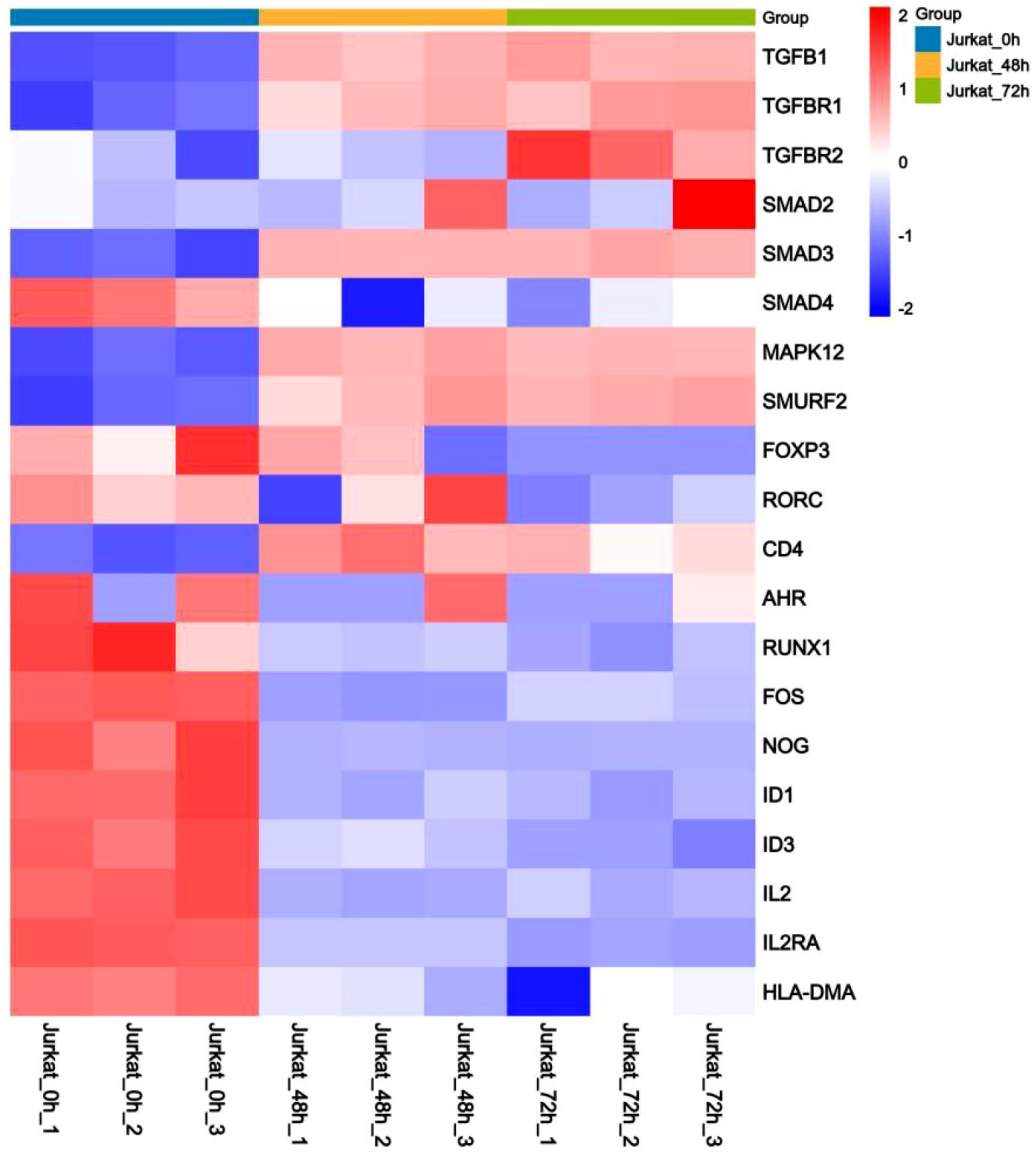
Heatmap of differentially expressed genes related to the TGF-β signaling pathway and Treg, Th17 cell differentiation. “Jurkat_0h” represents the 0 h control cells, “Jurkat_48h” represents the 48 h TPx-treated cells, and “Jurkat_72h” represents the 72 h TPx-treated cells. In the image, red color indicates genes encoding proteins with relatively high expression, while blue color indicates genes encoding proteins with relatively low expression.

Further, 20 differentially expressed genes associated with the TGF-β signaling pathway and Treg/Th17 cell differentiation were selected to construct a heatmap of differential genes, comparing the differences between the 0 h control cells, 48 h TPx-treated cells, and 72 h TPx-treated cells. As shown in **Figure 8**, compared with the 0 h control cells, the relative expression levels of differentially expressed genes such as *TGF-β*, *TGF-βR1*, *TGF-βR2*, and *SMAD3* were higher in the 48 h and 72 h TPx-treated cells, while the change in *SMAD2* was not significant. Notably, with increasing exposure time to *T. solium metacestode* TPx protein, the relative expression levels of the differential genes *TGF-β, TGF-βR1*, and *SMAD3* showed an increasing trend. Compared with the 0 h control cells, the relative expression levels of differential genes such as *SMAD4, FOS*, and *RUNX1* were lower in the 48 h and 72 h TPx-treated cells. No significant changes in *RORC(γt)* and *FOXP3* were observed in the 48 h TPx-treated cells; however, both genes showed lower relative expression levels in the 72 h TPx-treated cells. Thus, TPx protein treatment of Jurkat cells for 48 h and 72 h can upregulate the TGF-β signaling pathway in Jurkat cells and may further regulate Treg/Th17 cell differentiation through transcription factors such as TGF-β, TGF-βR1, SMAD3, FOXP3, and RORC(γt).

### 3.3 The expression levels of proteins and phosphorylated proteins associated with Treg and Th17 cell differentiation that are involved in the TGF/Smad signaling pathway

The differential transcriptomic results for Jurkat cells induced by TPx protein indicate that key proteins such as TGF-β1, TGF-βR2, Smad4, Smad3, FOXP3, and RORC(γt) in the TGF-β/Smad signaling pathway may be involved in the processes that lead to Treg/Th17 cell imbalance. Therefore, western blot analysis was used to detect the expression levels of these proteins during the dynamic changes in Treg/Th17 cell balance.

**Figure 9:**
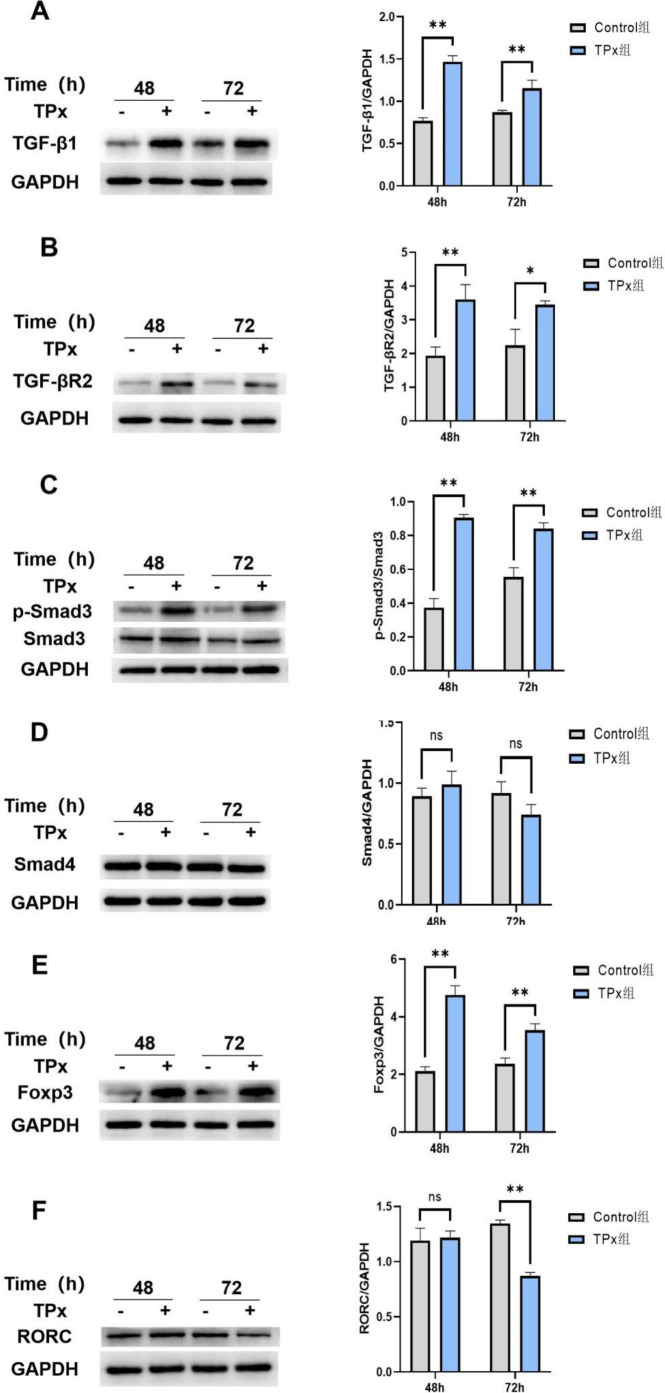
Effect of TPx protein on the expression of proteins related to the TGF-β/Smad signaling pathway (n = 3, x ± S). **(A)** TGF-β1/GAPDH ratio; **(B)** TGF-βR2/GAPDH ratio; **(C)** p-Smad3/Smad3 ratio; **(D)** Smad4/GAPDH ratio; **(E)** Foxp3/GAPDH ratio; **(F)** RORC(γt)/GAPDH ratio; * means *P* < 0.05; ** means *P* < 0.01; ns means no significant difference.

The results are shown in **Figure 9**. Compared with those in the control cells, the protein expression levels of TGF-β1, TGF-βR2, and p-Smad3 were significantly increased after 48 h and 72 h of TPx protein induction (*P* < 0.05). After 48 h of TPx protein induction, Smad4 protein expression showed an upregulated trend, but the difference was not significant (*P* > 0.05). Compared with the 72 h control cells, there was no significant change in Smad4 protein expression after 72 h of induction (*P* > 0.05).

Compared with those in the control cells, the FOXP3 protein expression levels were significantly increased after 48 h and 72 h of TPx protein induction (*P* < 0.05). After 48 h of TPx protein induction, the change in RORC(γt) protein levels was not significant (*P* > 0.05). However, the RORC(γt) protein expression level was significantly decreased after 72 h of induction (*P* < 0.05).

## 4 Discussion

Cysticercosis poses a greater threat than *T. solium* infection, and is primarily distributed in low- and middle-income developing countries. However, with globalization, some developed countries have also gradually reported imported cases (20, 21). Advances in molecular biology and immunology have increased our understanding of immune defense responses and immunopathological mechanisms related to parasitic infections in recent years. After invading the host, parasites avoid elimination by using various mechanisms to evade the host’s innate immune system. However, the host can suppress parasitic growth by inducing antigen-specific T cell differentiation into effector cells (22). For parasites to survive, they must evade this acquired immune defense of the host (23). *T. solium metacestode* can induce varying degrees of immune responses in the host at different infection stages, with the parasitic location and infection severity closely related to changes in T lymphocyte subsets (24).

Treg cells are significantly increased in the peripheral blood and cerebrospinal fluid of patients with NCC, leading to immune dysfunction characterized by an immunosuppressive state (25–27). In this study, we observed that *T. solium metacestode* TPx protein can inhibit the differentiation of CD4^+^CD25^+^CD127^−^ Treg cells at 24 hours, with no significant effect on the differentiation of CD4^+^IL-17A^+^ Th17 cells, resulting in no notable difference in the Treg/Th17 cell ratio relative to the control cells. However, at 48 and 72 hours, TPx protein significantly induced the differentiation of CD4^+^CD25^+^CD127^−^ Treg cells and inhibited the differentiation of CD4^+^IL-17A^+^ Th17 cells, leading to an imbalance between Treg and Th17 cells. This imbalance manifests as an inhibitory immune response predominantly involving Treg cells, which may represent a true “window” that favors the evasion of the host’s immune response by *T. solium metacestode* during the later stages of infection. Through GSEA, we discovered that *T. solium metacestode* TPx protein can activate the TGF-β/Smad signaling pathway in human Jurkat T lymphocytes at both 48 h and 72 h of exposure, and multiple related transcription factors such as TGF-β, TGF-βR1, TGF-βR2, and Smad3 were upregulated under the influence of TPx protein. These findings collectively suggest that the TGF-β/Smad signaling pathway may be a key pathway through which the TPx protein of *T. solium metacestode* regulates host immune responses.

In this study, it was found that *T. solium metacestode* TPx protein can upregulate the expression of TGF-β1, TGFβR2, and p-Smad3 proteins at 48 h and 72 h, activating the TGF-β/Smad signaling pathway. Additionally, the expression of the key transcription factor Foxp3, which regulates Treg cell differentiation, showed an upward trend at both 48 h and 72 h, while the expression of RORC (γt), a key transcription factor for Th17 cell differentiation, showed a downward trend at 72 h. This suggests that *T. solium metacestode* TPx protein can regulate Foxp3 expression and induce Treg cell differentiation by activating the TGF-β/Smad signaling pathway at 48 h and 72 h. With increased duration of stimulation, its inhibitory effect on Th17 cells gradually increases, significantly suppressing Th17 cell differentiation at 72 h. This induces a reversal in the Treg/Th17 cell ratio imbalance, leading to a predominance of Treg cells. This shift benefits *T. solium metacestode* by aiding its evasion of the host’s immune response in the later stages of infection, potentially resulting in the parasite’s escape from immune attack and establishment of chronic infection. In the present study, we observed a concurrent downregulation of both Foxp3 and RORC (γt) mRNA expression at the transcriptome level; however, at the protein level, Foxp3 expression was notably upregulated. This seemingly paradoxical phenomenon may precisely reveal a sophisticated and complex mechanism through which the TPx protein regulates the host immune response. The transient decrease in Foxp3 mRNA coupled with the progressive accumulation of its protein reflects a dynamic and asynchronous regulatory phase during Treg cell differentiation. The expression of key transcription factors such as Foxp3 is subject to stringent, multi-layered regulation. Although its mRNA level decreases, its protein translation efficiency, stability, or post-translational modifications (e.g., phosphorylation, ubiquitination) may be significantly influenced by the TPx protein. For instance, the TPx protein may stabilize Foxp3 protein or inhibit its degradation via the ubiquitin-proteasome pathway, potentially through the TGF-β/Smad signaling axis or other unidentified pathways, thereby leading to its accumulation at the protein level despite reduced mRNA expression. In this study, we also found that *T. solium* metacestode TPx protein can downregulate the expression of Runt-related transcription factor 1 (RUNX1) at 48 h and 72 h. RUNX1 can cooperate with Foxp3 to promote Treg cell differentiation and also bind to RORγt to drive Th17 cell differentiation (28, 29). This suggests that downregulating RUNX1 may favor steering the differentiation program toward a Foxp3-dominated Treg pathway rather than an RORγt-dominated Th17 pathway. This provides clear direction and rationale for our subsequent investigations into the underlying mechanisms.

During various stages of parasitic infection, T cell immune response-related signaling pathways can participate in cell differentiation, inflammation, and immune response through interactions among signaling molecules. Transcriptomic analysis in this study revealed that *T. solium metacestode* TPx protein induces downregulation of the MAPK signaling pathway and the phosphatidylinositol 3-kinase (PI3K)/AKT signaling pathway in Jurkat cells. The MAPK pathway mainly comprises four sub-pathways: extracellular signal-regulated kinase (ERK) 1/2, c-Jun N-terminal kinase (JNK), p38 MAPK, and ERK5 (30). Phosphorylation of p38 MAPK, JNK, and ERK can increase the production of inflammatory factors, thereby exacerbating the inflammatory response (31). Mitogen-activated protein kinase (MEK) is a specific activator of ERK. Studies have shown that in mouse splenic CD4^+^ T cells, blocking the MAPK/ERK signaling pathway with MEK siRNA increases TGF-β-induced Foxp3 expression and enhances Treg cell differentiation, while reducing RORγt expression and Th17 cell differentiation. This suggests that the MAPK/ERK signaling pathway promotes differentiation of CD4^+^ T cells into Th17 cells while inhibiting Treg cell differentiation (32). The PI3K/AKT signaling pathway plays a crucial role in inflammatory immune cascades. Inhibition of the PI3K/AKT signaling pathway can impair the inflammatory response. Studies indicate that extracellular vesicles (EVs) from *T. solium metacestode* contain metabolites that negatively regulate the PI3K/AKT signaling pathway, promoting macrophage apoptosis. This may be a significant mechanism by which *T. solium metacestode* exerts immunosuppressive effects and causes asymptomatic infection in the early stages of NCC (33).

In summary, the results of this study indicate that the TPx protein can inhibit Treg cell differentiation at 24 hours, with no significant effect on Th17 cell differentiation. However, after 48 and 72 hours of culture, it can induce Treg cell differentiation, inhibit Th17 cell differentiation, and induce Treg/Th17 cell imbalance, resulting in a suppressive immune response dominated by Treg cells. Transcriptomic analysis of Jurkat cells before and after exposure to *T. solium metacestode* TPx protein revealed that the TGF-β/Smad signaling pathway can be activated at both 48 and 72 hours. Western blotting confirmed that the expression of key transcription factors TGF-β1, TGF-βR2, and p-Smad3 proteins involved in Treg and Th17 cell differentiation significantly increased at 48 and 72 hours, indicating that the TGF-β/Smad signaling pathway can be activated by the TPx protein. The expression level of Foxp3, a key transcription factor for Treg cell differentiation, significantly increased after 48 and 72 hours of induction, while the expression level of RORC(γt), a transcription factor related to Th17 cell differentiation, significantly decreased after 72 hours of induction but showed no significant change at 48 hours. This suggests that *T. solium metacestode* TPx protein may further regulate the expression levels of Foxp3 and RORC(γt) through the TGF-β/Smad signaling pathway, thereby inducing host Treg/Th17 cell imbalance. Taken together, these findings suggest that *T. solium metacestode* TPx protein may primarily induce host Treg/Th17 cell imbalance through the TGF-β/Smad signaling pathway, providing important experimental data for further verifying the role of the TGF-β/Smad signaling pathway in the immune regulation of cysticercosis. The findings offer valuable insights to elucidate the molecular mechanisms involved in the pathogenesis of cysticercosis and provide new strategies for the prevention and treatment of this disease.

## 5 Conclusion

The *T. solium metacestode* TPx protein induces early (24 h) reduction in Treg cell differentiation and late (48 h, 72 h) Treg/Th17 cell imbalance in human Jurkat cells, leading to an inhibitory immune response predominantly involving Treg cells. The dynamic immunodifferentiatory mechanism underlying this phenomenon is associated with the TGF-β/Smad signaling pathway. *T. solium metacestode* TPx protein can activate this pathway to further regulate the expression levels of key transcription factors Foxp3 and RORC(γt) involved in Treg and Th17 cell differentiation, thereby affecting the balance between Treg and Th17 cells.

## Data Availability

The datasets presented in this study can be found in online repositories. The names of the repository/repositories and accession number(s) can be found below: https://data.4tu.nl/private_datasets/wJMc9JOSVpHcNsBCKFajC_BTBaRlg--YM5fy-CrEnUs.
